# Assessing the fidelity of a peer-led chronic pain management program (PAP)

**DOI:** 10.1186/s13063-021-05599-6

**Published:** 2021-09-20

**Authors:** Mimi M. Y. Tse, Shuk Kwan Tang, Shamay Ng, Yajie Li, Daphne Sze Ki Cheung, Rick Yiu Cho Kwan

**Affiliations:** 1 School of Nursing, The Hong Kong Polytechnic University, Kowloon, Hong Kong; 2 Department of Rehabilitation Sciences, The Hong Kong Polytechnic University, Kowloon, Hong Kong; 3 School of Nursing, The University of Hong Kong, Pokfulam, Hong Kong

**Keywords:** Intervention fidelity, Peer-led pain management program (PAP), Clustered randomized controlled trial, Peer volunteer (PV)

## Abstract

**Background:**

Intervention fidelity is the core component of a well-designed clinical trial and processes that are used to ensure that the study intervention is delivered as planned. It affects the design and implementation of a study as well as the analysis of the results and interpretation of the findings. The objectives of this study are (a) to describe the methods of assessing the intervention fidelity used in the peer-led chronic pain management program (PAP) and (b) to report the findings on the PAP’s intervention fidelity.

**Methods:**

To optimize fidelity to the intervention, we used various strategies and measured them in a continuous process using several different approaches: (a) peer volunteer (PV) training workshop, (b) biweekly meetings with the research team, (c) a detailed teaching manual, (d) a fidelity checklist, (e) on-site visits and direct observations, and (f) semi-structured interview.

**Results:**

The PVs’ attendance was high, and most of them achieved a high level of implementation in following the fidelity checklist. As part of a large clustered RCT, the fidelity assessment was carried out to help determine the effectiveness of the intervention.

**Conclusions:**

Overall, the PVs successfully delivered the intervention, and the results of the study indicate the effectiveness of the PAP.

**Trial registration:**

ClinicalTrials.govNCT03823495. Registered on 30 January 2019.

**Supplementary Information:**

The online version contains supplementary material available at 10.1186/s13063-021-05599-6.

## Background

It is regarded that a well-designed randomized controlled trial is the gold standard for the development of effective interventions in healthcare. The effects of an intervention can be impacted by insufficient implementation, which may also lead to inaccurate explanations of the findings of the study [1]. Intervention fidelity is regarded as an important component of an intervention, as it affects the design and implementation of a study as well as the analysis of the results and interpretation of the findings. Fidelity is defined as “the methodological strategies applied to monitor and ensure the reliability and validity of behavioral interventions” [2]. It is a core component of a well-designed clinical trial and processes that are used to ensure that the study intervention is delivered as planned [3–7]. Intervention fidelity requires careful consideration of the design of the study, the development of the intervention protocol, and the training of the research staff [3, 6]. The lack of a method to assess the fidelity of an intervention may lead to bias in the findings of a study and to uncertainty over whether the results were affected by the level of infidelity to the intervention or by the ineffectiveness of the intervention [8].

Process fidelity and content fidelity have been reported as the different components of intervention fidelity [1]. There are various strategies for assessing fidelity, such as via an intervention manual, direct observation, and videotapes or audiotapes of the implementation of the intervention [2, 6]. Studies have pointed out that it is necessary to develop fidelity assessment tools suitable for a particular intervention [9].

The study that we conducted was a clustered randomized control trial entitled *Effectiveness of a peer-led pain management program in relieving chronic pain and enhancing pain self-efficacy among older adults*. The intervention that we designed for our study was a peer-led chronic pain management program (PAP) led by a peer volunteer (PV) using a teaching manual, and the group that received this intervention was compared with a group that received the usual care. The Stanford Chronic Disease Self-Management Program and the chronic pain self-management program developed by Ersek et al. were used as our theoretical framework [10]. The focus of our study was on the ability to manage pain and pain-related symptoms, the treatment of pain, and the physical and psychosocial consequences and lifestyle changes inherent in living with a chronic pain condition.

The study was carried out in 21 government-subsidized nursing homes in Hong Kong. Eligible participants were randomly allocated to either the experimental group or the control group according to a computer-generated list. In the experimental group, nursing home residents underwent one 1-h learning session per week for 12 weeks, led by peer volunteers (PVs). Each session began with 20 min of physical exercise performed under the supervision of the PVs. This was followed by 30 min of pain management education, which covered such topics as pain situations, the impacts of pain, the use of drug and non-drug strategies for managing pain, and demonstrations and return demonstrations of various non-drug pain management techniques. At the end of each session, the PVs would help the participants put together a portfolio on the activities of the day, to help them recall what they had learned.

To more accurately interpret the findings of the study, the aims of this study are (a) to describe the methods of assessing the intervention fidelity used in the PAP and (b) to report the findings on the PAP’s intervention fidelity.

## Ethical considerations

Ethical approval and the written informed consent of all the PVs were obtained prior to the start of the PAP.

## Statistical analysis

The Statistical Package for the Social Sciences (SPSS) version 23 (IBM Corporation, Armonk, NY) was used to handle and analyze the data. Descriptive statistics were used to present the results. After the interview, qualitative data on the contents of the interview were analyzed.

## Results

### Methods of assessing the intervention fidelity

This study discusses the assessment of the fidelity to the intervention that was employed in the clustered RCT entitled *Effectiveness of a peer-led pain management program in relieving chronic pain and enhancing pain self-efficacy among older adults*.

Volunteers were recruited and trained to lead the PAP for older adults. These volunteers were recruited from an institute hosted by a local university in HK based on the following criteria:

1. Older than 55 years old
2. Scored over 6 in the Abbreviated Mental Test (AMT)
3. Be willing to lead the PAP in a nursing home

After the volunteers were selected, they were required to attend several workshops and pass an exit exam before they participated in the PAP and led the intervention provided for the experimental group.

### Intervention fidelity

To optimize fidelity to the intervention, various strategies and measures were used in a continuous process as follows:

- PV training workshops. Four 2-h training workshops were conducted over 2 weeks. The topics of the workshops are shown in Table 1: The materials used in the workshops were uploaded to a free online platform (Google Drive). Volunteers who would like to review were allowed to access the materials at any time. The training methods include dialectic lecturing (group), small group discussions, case sharing, and demonstrations and return demonstrations (individual) on non-pharmacological approaches to pain management. Both group-based activity and individual-based consultation are involved. The return demonstrations were designed as individualized coaching sessions to ensure that the skills were mastered. After the workshops, an exit test was prepared to guarantee the quality of the learning.
- Biweekly meetings with the research team. To discuss and review the cases and receive “booster” sessions of pain management education.
- A detailed teaching manual. For the PVs to follow and lead the PAP.
- A fidelity checklist (Additional file 1). To guide PVs when carrying out the PAP. The fidelity checklist set out the implementation of the PAP in terms of four levels: low/not observed, observed to a small degree, observed to a medium degree, and high implementation, which was developed based on a recent similar study. PVs were required to demonstrate 95% implementation at a high level.
- On-site visits and direct observations. Each PV was visited three times in 12 sessions and observed directly by the research team. The PVs were randomly selected for observation using an online randomizer (https://www.random.org/), as guided by the fidelity checklist. Their attendance was also measured.
- Semi-structured interview. All the PVs were interviewed after the intervention, following an interview guide (Additional file 2). They were asked about their experiences and feedback in leading the PAP.

**Table 1:** Topics of workshops for selected volunteers

	Topic
1.	What a peer is
2.	Communication skills
3.	Client safety and confidentiality
4.	Managing crises and emergencies
5.	Motivational strategies to enhance the compliance of the participants
6.	Demonstrations on the use of the teaching manual, i.e., “I can do it” and various non-pharmacological practices

#### Demographic characteristics of the PVs

A total of 46 peer volunteers were selected to lead the pain management program. More than 73% of them were female. All the PVs were over the age of 80, and 73.9% were married. More than half of the PVs had a university education or above. The occupations of the PVs included laborers, technicians, clerks, and housewives. Twenty-six percent of the PVs had a chronic disease, and of this group, half had hypertension. Sixty-seven percent of the PVs experienced chronic pain, with the legs being the most reported site of the pain. Their average pain score was 2.37 on a 10-point scale. Details are presented in Table 2.

**Table 2:** Demographic characteristics of the PVs

Variables	***N*** (%)	Mean (***SD***)
Gender		
F	34 (73.9)	
M	12 (26.1)	
Age		60.95 (5.07)
< 60	22 (64.7)	
60–70	22 (64.7)	
71–80	2 (4.3)	
Marital status		
Single	4 (8.7)	
Married	34 (73.9)	
Divorced	5 (10.9)	
Widowed	3 (6.5)	
Education level		
Primary School	1 (2.2)	
Secondary School	17 (37.0)	
University or above	28 (60.9)	
Occupation		
Physical laborer	2 (4.3)	
Technician	23 (50.0)	
Housewife	3 (6.5)	
Clerk	17 (37.0)	
Any chronic illness		
Yes	12 (26.1)	
No	34 (73.9)	
Chronic diseases		
Heart disease	2 (4.3)	
Diabetes	1 (2.2)	
Hypertension	6 (13.0)	
Cancer	2 (4.3)	
Cataract	2 (4.3)	
Stroke	1 (2.2)	
Arthritis	1 (2.2)	
Other chronic diseases	1 (2.2)	
Chronic pain		
Yes	31 (67.4)	
No	15 (32.6)	
Pain sites		
Head	6 (13.0)	
Shoulders	8 (17.4)	
Arms	9 (19.6)	
Back	10 (21.7)	
Legs	17 (37.0)	
Pain intensity (lowest)		2.37 (2.04)
Has voluntary experience	40 (87.0)	
Self-rated confidence in volunteering		78.7 (16.3)
Self-rated pain knowledge		40.0 (20.5)
Pain knowledge score		86.1 (10.6)

#### Attendance as determined by on-site visits

**Fig. 1:**
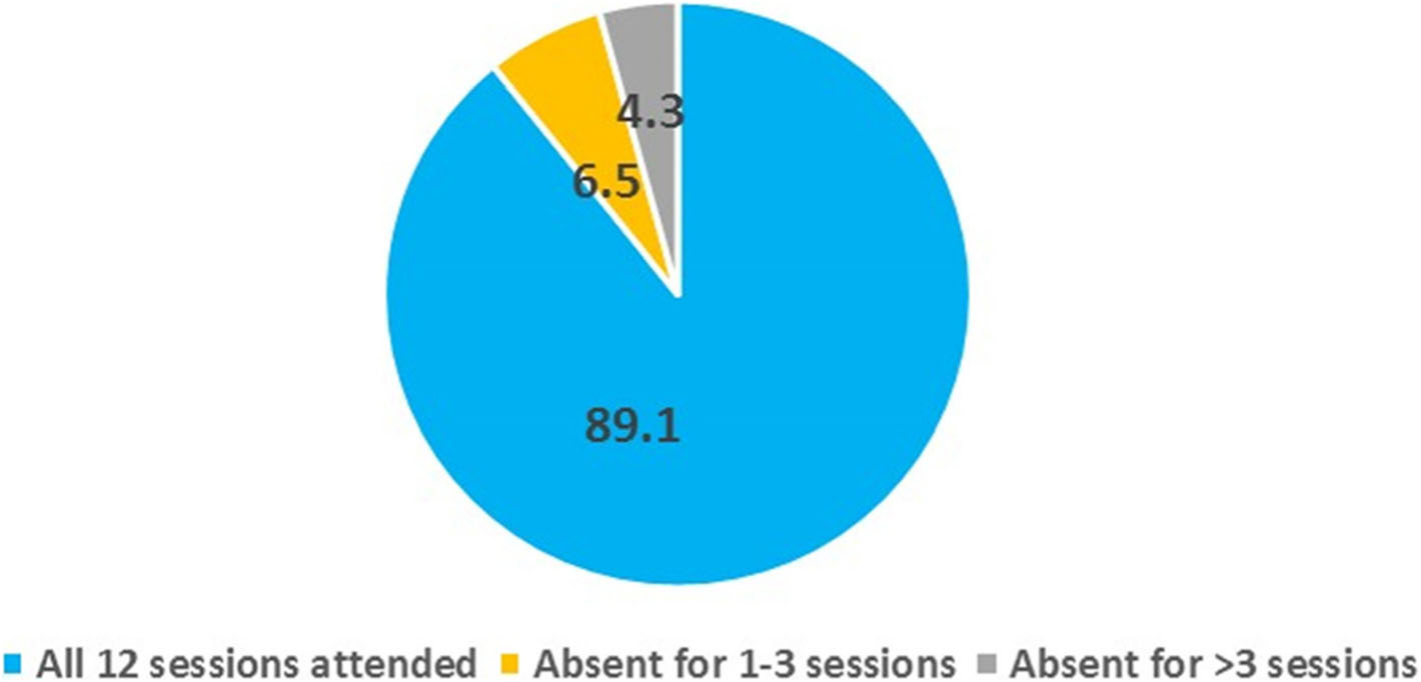
Attendance of the peer volunteers

The PVs’ attendance during the program was recorded, and the figures are reported in Fig. 1. Approximately 90% of the PVs took part in all 12 sessions. Only two PVs were absent for more than 3 sessions.

#### The PVs’ level of implementation (following the fidelity checklist)

In total, 46 PVs were observed 138 times (each PV was observed 3 times). Their level of implementation is shown in Table 3. In most of the required behaviors, a high-level implementation was achieved. The sole exceptions were in the following areas: “Was the PV organized and familiar with the teaching manual in teaching the PAP?” and “Did the PV complete all parts of the session?”, where the research team observed a medium level of implementation of 3.6% and 0.7%, respectively. Of the 11 items in the checklist, a high level of implementation was achieved in 9 items.

**Table 3:** PV: level of implementation (fidelity checklist)

PV’s behavior	Level of implementation observed, *N* (%)			
	Low/not observed	Small	Medium	High
**General observations on the organization of the teaching**				
1. Were the materials ready for each activity?				138 (100)
2. Was the PV organized and familiar with the manual for teaching the PAP?			5 (3.6)	133 (96.4)
3. Was the PV teaching according to the teaching manual?				138 (100)
4. Did the PV complete all parts of the session?			1 (0.7)	137 (99.3)
5. Did the PV provide opportunities for the participants to respond?				138 (100)
6. Did the PV use session activities?				138 (100)
**On encouragement**				
1. Did the PV use the principle of pain self-management as laid out in the teaching manual?				138 (100)
2. Did the PV teach the residents to maintain functional activities, e.g., grooming and walking even in situations of pain?				138 (100)
3. Did the PV explain to the participants the importance of using various strategies that can help to relieve pain and pain-related situations?				138 (100)
4. Did the PV practice non-pharmacological strategies with the participants?				138 (100)
5. Did the PV encourage the participants to self-practice the strategies for pain relief that they learned in class?				138 (100)

#### Semi-structured interview

The comments and feedback of the PVs, given in a semi-structured interview, are shown in Table 4. The PVs felt a sense of satisfaction and meaningfulness in leading this program, as it was effective in helping the nursing home residents to reduce their pain. They also gave some suggestions on how to improve this program, including by “adding more practical strategies,” “holding more workshops for PVs during the program to share their experiences and learn,” and “taking the comments of nursing home staff into consideration.”

**Table 4:** Comments and feedback in the semi-structured interview

***A. Please describe your experience in leading the pain management program***	
1. It was an interesting and meaningful experience to lead this program. 2. I was appreciated by the nursing home residents. 3. The nursing home residents said that they liked us and expected that we would visit them more often.	
***B. Please share your perception of the benefits of the whole program***	
1. It seems that my pain was also reduced when teaching the nursing home residents. 2. Helping others makes me feel happy and is meaningful to me. 3. This program is quite effective, as the elderly said that they feel less pain and are happier and less lonely. 4. I feel satisfied that I can help others.	
***C. Please share the limitations and barriers that you encountered in teaching the pain management methods***	
1. Some pain management strategies were difficult to apply in the nursing homes, such as applying hot/cold compresses, as the nursing homes lacked sufficient materials. 2. There was a lack of feedback from nursing home staff; their comments are also important.	
***D. Do you have any suggestions on how to further improve the pain management program?***	
1. It would be better to add more practical strategies. As the program is designed for elderly people living in nursing homes, the physical and environmental limitations need to be taken into consideration. 2. The research team could arrange to hold more workshops for us PVs during the program, so that we can share our experiences and learn from other PVs.	

## Discussion

The focus of this report is on the assessment of the fidelity of the intervention that we designed in our previous study, *Effectiveness of a peer-led pain management program in relieving chronic pain and enhancing pain self-efficacy among older adults*. The PVs’ attendance was high, and most of them achieved a high level of implementation in following the fidelity checklist.

Our study is consistent with a study that addresses and monitors fidelity [11], of which, programs and components must be measured. Indeed, addressing intervention fidelity in terms of enhancement, assessment, and reporting becomes particularly important for complex health behavior change interventions [12]. A fidelity assessment is essential to maintain the internal validity of a study and to ensure that a fair comparison is made of the results of the intervention and control settings [13]. The fidelity assessment is also related to external validity [14]. Enough information about the methodology, fidelity, and effectiveness of all relevant components of an intervention is important for effective implementation [15, 16].

In our study, the selection and training of PVs in our program improved its internal and external validity. The PVs that we selected to lead this pain management program were from the institute hosted by the university. To become a member of the institute, an applicant should be older than 50. Those wishing to join need to fill in an application form and pay an annual membership fee. The executive officers of the institute maintain close contact with all members and know them for long enough to be able to judge whether individuals have the mental/cognitive capacity to serve as PVs. The institute is an official organization and was an excellent source from which to select volunteers for this program, with the number of qualified members facilitating the process of selecting PVs who satisfied our criteria.

The fact that there are more females than males in our PV group, it is noted that females are more willing to act as volunteers than their male counterparts, and this may reflect that more females are suffering from pain. A higher prevalence of pain in women has been widely reported in epidemiological studies [17]. Cimas et al. [18] examined over 61,000 population of 50 years, from fourteen European countries, and found an overall prevalence of chronic pain of 35.7%. The prevalence of pain was significantly higher in women than in men in all countries in Cimas et al.’s study.

The theoretical framework developed by Ersek et al. was referenced in designing the workshops for the PVs [10]. Various teaching methods were employed, including dialectic lecturing (group), small group discussions, case sharing, and demonstrations and return demonstrations (individual) of non-pharmacological practices. The instructional model is largely a group-based one. However, the research team was available for individual consultations, and the return demonstration involved individualized coaching. The individualized activities gave the PVs opportunities to put their theoretical knowledge into practice, with the research team present to find their problems and correct their mistakes in time. This allowed the PVs to better understand the program and to be well-prepared to lead the program in the nursing homes. Ensuring that the PVs master the strategies of pain management is vital to improve the validity of the intervention as well as fidelity to the intervention. These were consistent with intervention fidelity addressing in terms of enhancement, assessment, and reporting [12].

Utilizing a comprehensive protocol can lead to greater consistency and precision in the delivery of an intervention and can enhance the interval validity of the intervention. In our study, a well-designed comprehensive teaching manual for PVs was prepared by the research team, to improve the validity of the intervention and achieve high intervention fidelity. Thus, our results showed a high attendance rate and implementation level. It has been noted that the absence of a comprehensive protocol may reduce the intervention fidelity rate, as PVs may be unfamiliar with the process of the program [15]. To this end, a well-designed comprehensive protocol achieves a balance between standardization to support adherence and internal validity, and flexibility to support competence and external validity.

The results of the assessment of the fidelity of our intervention indicate the effectiveness of our program. Previous studies have pointed to an insufficiency of studies assessing the fidelity of geriatric care interventions, with most fidelity assessment studies having been carried out in the area of psychiatric rehabilitation [5, 9, 15]. Our study contributes to the assessments of fidelity in the area of geriatric programs.

It has been pointed out that in a fidelity assessment, it is important to collect data from multiple sources, to minimize reporting bias. Yet, we only collected data from the perspective of the PVs, and this constituted one of the limitations of the present study. In addition, the perspectives of other groups involved in this study, including nursing home staff and nursing home residents, should also be considered. In addition, as indicated by a previous study [15], the differences between perspectives and response characteristics (i.e., direct observations, semi-structured interviews) should be calculated and taken into consideration, to better understand and explain the results.

## Conclusion

As part of a large clustered RCT, the fidelity assessment was carried out to help determine the effectiveness of the intervention. The results of this fidelity assessment are encouraging and significant for studies of the fidelity of geriatric interventions.

## Supplementary Information

**Additional file 1.** Fidelity checklist.

**Additional file 2.** Instruction for the semi-structured interview.

## Data Availability

The datasets used and/or analyzed during the current study are available from the corresponding author on reasonable request.

## Abbreviations

AMT: Abbreviated Mental Test
PAP: Peer-led chronic pain management program
PV: Peer volunteer
RCT: Randomized controlled trial
SPSS: Statistical Package for the Social Sciences
