# Supplementary material for: Assessing the fidelity of a peer-led chronic pain management program (PAP)
Source: Trials. 2021 Sep 20;22:644. doi: 10.1186/s13063-021-05599-6 (PMC8454105; doi:10.1186/s13063-021-05599-6)
Supplement: Supplementary file 1 — Additional file 1. Fidelity checklist. [file 13063_2021_5599_MOESM1_ESM.docx]

Additional file 1. Fidelity checklist

PV’s name _______________ Date _________________

Observer’s name ____________________

| **PVs behavior** | **Level of implementation** | | | |
| --- | --- | --- | --- | --- |
|  | Low/Not observed | Observed to a small degree | Observed to a medium degree | High implementation |
| **General observations on the organization of the teaching** |  |  |  |  |
| Were the materials ready for each  activity? |  |  |  |  |
| Was the PV organized and familiar with the teaching manual in teaching the |  |  |  |  |
| Was the PV teaching according to the teaching manual? |  |  |  |  |
| Did the PV complete all parts of the session? |  |  |  |  |
| Did the PV provide opportunities for the participants to respond? |  |  |  |  |
| Did the PV use session activities? |  |  |  |  |
| **On encouragement** |  |  |  |  |
| Did the PV use the principle of pain self- management as laid out in the teaching  manual? |  |  |  |  |
| Did the PV teach the residents to maintain functional activities, e.g., grooming and walking even in situations of pain? |  |  |  |  |
| Did the PV explain to the participants the importance of using various strategies that can help to relieve pain and pain-related situations? |  |  |  |  |
| Did the PV practice non-pharmacological strategies with the participants? |  |  |  |  |
| Did the PV encourage the participants to self-practice the strategies for pain relief that were learned in class? |  |  |  |  |
