# Supplementary material for: Assessing the fidelity of a peer-led chronic pain management program (PAP)
Source: Trials. 2021 Sep 20;22:644. doi: 10.1186/s13063-021-05599-6 (PMC8454105; doi:10.1186/s13063-021-05599-6)
Supplement: Supplementary file 2 — Additional file 2. Instruction for the semi-structured interview. [file 13063_2021_5599_MOESM2_ESM.docx]

Additional file 2. Instruction for the semi-structured interview

1. Please describe your experience in leading the pain management program
2. Please share your perception of the benefits of the whole program
3. Please share the limitations and barriers that you encountered in teaching the pain management methods
4. Do you have any suggestions for improving the pain management program?
